# Sebaceous Tumors and Their Cure

**Published:** 1897-04

**Authors:** A. Bethune Patterson

**Affiliations:** Atlanta, Ga.


### SEBACEOUS TUMORS AND THEIR CURE.

By A. BETHUNE PATTERSON, M.D.,

ATLANTA, GA.

While in general practice I was often called upon to remove sebaceous tumors—in common parlance, "wens"—and, as is well known, the dissecting out the sac is a tedious and often a bloody operation, and is by no means as frequently resorted to as these common-place tumors would suggest. Authors on surgery have insisted on the entire removal of the secreting wall, as a portion left has often resulted in the reforming of the cystoma. Injections of iodine and carbolic acid have often proved successful. The following case will illustrate the conditions which led me in an accidental way to effect a cure:

F. W., age 60; a large sebaceous tumor about the size of an orange, occupying a position a few inches below the shoulder joint, and encroaching upon the outer margin of the deltoid muscle. On the summit of the growth three small ulcers had formed in close proximity and extending into the cyst cavity. With a view of disinfecting these ulcers, I injected into them peroxide of hydrogen. Effervescence immediately began, forcing out a small quantity of sebaceous matter. A free incision about one inch was made and the contents squeezed out. I thoroughly cleansed the sac with peroxide and applied an antiseptic dressing, which was removed in five days. My patient was so pleased with the painless and rapid cure he returned two months later to have a similar growth operated upon situated upon the scapula. An incision was made about an inch long through the skin and sac wall, after cocainizing by hypodermic injection, and I squeezed out the sebaceous matter and cleansed the sac with repeated injections of the peroxide; antiseptic dressing remained eight days. The latter operation was performed nine months ago, and, as with the first, there has been no reforming of the sebaceous matter.

I have used for several years the peroxide of hydrogen in my eye, ear, and throat work, and find in impacted cerumen, when hard and tenaceous, it disintegrates the mass quicker than soda or anything else I have tried. The wax becomes crumbly and is then easily removed by injections of warm water.

*202 Norcross Building.*

